# Comparison of clinical characteristics and healthcare resource use of pediatric chronic and non-chronic critically ill patients in intensive care units: a retrospective national registry study

**DOI:** 10.3389/fped.2023.1194833

**Published:** 2023-06-26

**Authors:** Chantal Grandjean, Marie-Hélène Perez, Anne-Sylvie Ramelet, The OCToPuS Consortium, Anne-Laure Lauria

**Affiliations:** ^1^ Pediatric Intensive and Intermediate Care Unit, Department Woman-Mother-Child, Lausanne University Hospital, Lausanne, Switzerland; ^2^ Institute of Higher Education and Research in Healthcare, Faculty of Biology and Medicine, University of Lausanne, Lausanne, Switzerland; ^3^ Faculty of Biology and Medicine, University of Lausanne, Lausanne, Switzerland

**Keywords:** health resources, chronic disease, chronic critical illness (CCI), critical care, intensive care units, pediatrics

## Abstract

**Introduction:**

Chronic critically ill patients (CCI) in pediatric intensive care unit (PICU) are at risk of negative health outcomes, and account for a considerable amount of ICU resources. This study aimed to (a) describe the prevalence of CCI children, (b) compare their clinical characteristics and ICU resources use with non-CCI children, and (c) identify associated risk factors of CCI.

**Methods:**

A retrospective national registry study including 2015–2017 data from the eight Swiss PICUs of five tertiary and three regional hospitals, admitting a broad case-mix of medical and surgical patients, including pre- and full-term infants. CCI patients were identified using an adapted definition: PICU length of stay (LOS) ≥8 days and dependence on ≥1 PICU technology.

**Results:**

Out of the 12,375 PICU admissions, 982 (8%) were CCI children and compared to non-CCI children, they were younger (2.8 vs. 6.7 months), had more cardiac conditions (24% vs. 12%), and higher mortality rate (7% vs. 2%) (*p *< 0.001). Nursing workload was higher in the CCI compared to the non-CCI group (22 [17–27]; 21 [16–26] respectively *p* < 0.001). Factors associated with CCI were cardiac (aOR = 2.241) and neurological diagnosis (aOR = 2.062), surgery (aORs between 1.662 and 2.391), ventilation support (aOR = 2.278), high mortality risk (aOR = 1.074) and agitation (aOR = 1.867).

**Conclusion:**

the results confirm the clinical vulnerability and the complexity of care of CCI children as they were defined in our study. Early identification and adequate staffing is required to provide appropriate and good quality care.

## 1. Introduction

Due to advances in critical care over time, survival in pediatric intensive care units (PICU) increases worldwide and results in numerous chronic critically ill (CCI) children (1, 2). Patients are considered CCI when they have history of a prolonged PICU length of stay (LOS), ongoing acute care needs, persistent multiorgan dysfunction, and dependence on life-sustaining technology (2, 3). Prevalence rate of patients with PICU LOS ≥14 days ranges between 6% and 14% (4, 5) with reported mortality rate between 8% and 20% (4, 5). Survivors are particularly prone to developping nosocomial infections (6, 7), delirium (8), poor neurologic and developmental outcomes (9) and poor quality of life (QOL) (10). These patients account for a considerable amount of PICU healthcare resources; 5% of patients with PICU LOS ≥19 days can use up to 40% of PICU bed-day occupancy (11). Most CCI children (85%) require mechanical ventilation and inotropic support (10). Life-sustaining technologies and complexity of care characterizing CCI children are associated with increased healthcare professionals’ workload and mortality (12, 13). Risk factors of prolonged PICU LOS include cardiorespiratory and neurological conditions, young age, and high mortality risk (11, 14, 15).

Early identification of CCI children is essential to plan individualized care, with the goal of optimizing long-term patient outcomes. Traditionally, these children were identified using PICU LOS only. However, a definition that includes clinical complexity criteria in addition to a PICU LOS may be more appropriate (2, 15), especially in units admitting a broad mix of children which is the case for PICUs in Switzerland. To date, studies reporting the clinical characteristics of CCI children using clinical complexity criteria in addition to PICU LOS are limited (2, 16). The extent of resources used by CCI children (using same criteria) compared to non-CCI children is not known. Therefore, the objectives of this national registry study were (a) to describe the prevalence of CCI children in Swiss PICUs; (b) to compare clinical characteristics, nursing workload, and PICU resources use between CCI and non-CCI children; and (c) to identify factors associated with CCI in PICU.

## 2. Methods

This study followed the Strengthening the Reporting of Observational Studies in Epidemiology reporting guidelines for cross-sectional studies (Supplemental Digital Content). The research protocol was approved by the Human Research Ethics Committees in Switzerland (project-ID: 2019-00944, 05.09.2019). Participants consent was waived, as data were anonymous.

### 2.1. Design and data sources

We performed a retrospective observational study of PICU hospitalizations between January 1, 2015 and December 31, 2017. Data were extracted from the administrative and clinical records of the Minimal Dataset of the Swiss Society of Intensive Care Medicine (SSMI), including data from the eight mixed PICUs/NICUs in Switzerland, five of them being from a tertiary referral hospital, and three from a regional hospital. Altogether, they account for 100 beds and approximately 5’000 annual admissions. They admit a broad case-mix including medical and surgical patients, as well as full-term and preterm infants.

### 2.2. Case identification

**Figure 1:**
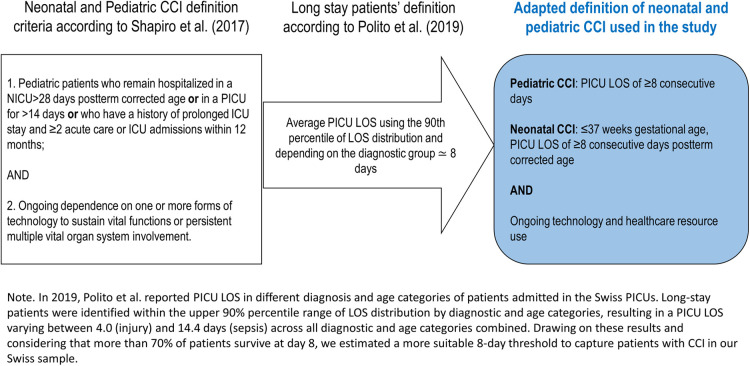
Adapted definition of neonatal and pediatric CCI.

CCI children were identified according to the following definition adapted from (2, 15) (Figure 1):

– Pediatric CCI (PCCI): from 1 month to 18 years, PICU LOS of ≥8 consecutive days, and ongoing technology and healthcare resource use
– Neonatal CCI (NCCI): ≤37 weeks gestational age, PICU LOS of ≥8 consecutive days postterm corrected age, and ongoing technology and healthcare resource use

Non-CCI children were children who did not meet the CCI criteria.

Exclusion criteria for CCI and non-CCI children: >18 years of age, transfers from another PICU.

Life-sustaining technology dependence was defined as per Shapiro’s definition and the criteria of the Swiss Society of Intensive Care Medicine to define technology, as follows: ongoing requirement of ≥1 of the following (a) invasive or non-invasive ventilation, or other ventilation (spontaneous breathing via endotracheal tube or tracheostomy, continuous positive airway pressure without inspiratory support or oxygen therapy); (b) intravenous (IV) medications (inotropic support, analgo-sedation, and other continuous drug infusions, except for iv maintenance fluids management); (c) dialysis; (d) Extracorporeal Membrane Oxygenation therapy (ECMO) (2).

Children were identified as CCI at PICU day 8 and were considered CCI until discharge, regardless of their requirement of life-sustaining technology throughout their remaining PICU stay.

### 2.3. Measurements

The demographic and clinical characteristics extracted from the dataset included: age, sex, PICU LOS, unplanned PICU admissions, PICU readmissions within 48 h, principal diagnosis, surgical interventions, mortality risk score, provenance before PICU admission and destination after PICU discharge, mortality in PICU, and therapeutic limitations (17).

PICU resources use included nursing workload, patient’s agitation status, PICU technologies and healthcare resources use. Nursing workload was measured using the validated Nine Equivalents of Nursing Manpower use Score (NEMS), including the following items, basic monitoring, intravenous medication, mechanical ventilation, supplementary ventilator care, single and multiple vasoactive medication, dialysis, and specific intervention in the ICU and outside the ICU (18). NEMS’ scores range between 0 and 56, with higher NEMS score indicating the more nursing manpower required during a shift or the ICU LOS. The NEMS was analyzed by computing a score at admission, one at discharge, and a score for the entire PICU LOS (average of all NEMS scores) scores were computed from the scores per shift divided by the PICU LOS for each admission records (≥24 h) (18). Patient’s agitation status was recorded each day with the Sedation Agitation Score (SAS) and presence of agitation was determined by a SAS score of 5 (agitated) or 6 (very agitated). PICU technologies and healthcare resources were measured using the frequency of utilization (during at least one shift throughout the PICU stay) and the duration of utilization (number of PICU days). Definitions of the variables are in presented in Table 1.

**Table 1:** Definition of variables.

Variable	Definition
Principal diagnostics	Using the Australian and New Zealand Paediatric Intensive Care (ANZPIC) registry of diagnostic code, they were further summarized as follows: respiratory, cardiac, neurological, injury, prematurity, sepsis, oncology, and other diagnosis (renal, digestive, hematologic and metabolic).
Surgical interventions	Were classified into eight categories: cardiac, abdominal, ear-nose-throat (ENT), neurological, orthopedic, thoracic, craniofacial surgeries, and post-procedure monitoring.
Therapeutic limitations	Including withholding treatments (not to start a particular treatment) or withdrawal of life-sustaining interventions (stopping a treatment that has been started)
Mortality risk	Measured using the Clinical Risk Index for Babies (CRIB II, range score: 0–27), the Pediatric Index of Mortality (PIM2, mortality prediction between 0 and 100%) between 0 and 16 years old, and the Simplified Acute Physiology Score (SAPS II, mortality prediction between 0 and 100%) for older than 16 years
PICU Technologies	Intravenous medications, invasive and non-invasive ventilation, other ventilation, multiple vasoactive medications, dialysis, and ECMO.
Healthcare resources	NEMS, patient isolation

### 2.4. Statistical analysis

The database included minimal missing data, except for the “withholding/withdrawal treatments” variable (≥10%). Because they were only documented when therapeutic limitation was indicated, they were treated as not applicable. No collinearity among variables was found (*r* > 0.6). Descriptive statistics were used to describe demographic and clinical characteristics of patients, and their PICU resource use. Categorical variables were summarized using frequency and percentage, and continuous variables using median and interquartile range. CCI and non-CCI groups were compared with Fisher’s exact test (nominal data) and Wilcoxon’s test (continuous data). Level of significance was two-sided α = 0.05. We performed bivariable and multivariable logistic regression models to identify the risk factors of CCI group, adjusted by centers. A conservative significance level of *p* = 0.20 was used to keep the variables in the multivariable model (19). Then, significant variables were kept in two multivariable logistic regression models: (1) to characterize CCI and non-CCI patients, (2) to identify risk factors of CCI. The first model investigated the following variables: principal diagnosis, surgical interventions, standardized mortality risk score, ventilation support, type of PICU admission, use of therapeutic limitations, agitation yes/no, total NEMS score at the different time points of measurement during the PICU hospitalization. The second model investigated significant variables in the bivariable models and that were available at the first day of PICU admission: principal diagnosis, surgical interventions, standardized mortality risk score, type of PICU admission. Adjusted odds ratios (aORs), standard error (SE) with 95% confidence intervals (CIs) were used to report the results. The analyses were performed using STATA version 15.0 (StatCorp College 214 Station, TX).

## 3. Results

### 3.1. Prevalence, demographic and clinical characteristics

We identified 12,736 admissions; 361 were excluded (>18 years *n* = 36 (0.3%), transferred from another PICU *n* = 325 (3%)). In total 12’375 PICU hospitalizations were analyzed, including 982 (8%) CCI children. Most of them was PCCI (87%). Demographic and clinical characteristics of the sample are presented in Supplementary material Table S1. The median LOS in the CCI group is significantly longer than the non-CCI (*p* < 0.001); 42% of them having a PICU LOS ≥14 days. CCI children compared to non-CCI were significantly younger, (2.8 months [0–36] and 6.7 months [0–60] respectively, *p* < 0.001), and had more unplanned PICU admissions (76% and 70%, *p* < 0.001). Provenance and destination after discharge were significantly different between the CCI and non-CCI groups (*p* < 0.001). More patients from the non-CCI group were admitted from the operating room than patients from the CCI group (49% and 44% respectively, *p* < 0.001), and these latter were discharged to the ward less often than non-CCI patients (66% and 56% respectively, *p* < 0.001). The principal diagnosis was significantly different between CCI and non-CCI groups (*p* < 0.001). Cardiac diagnoses were twice as frequent in the CCI group (231, 24%) as in the non-CCI group (1 325, 12%). Type of surgical interventions was significantly different between CCI and non-CCI groups (*p* < 0.001). Nearly half of CCI children had cardiac surgery (47%) vs. 26% in non CCI. Mortality risk of CCI children who were less than 16 years old (PIM2) was significantly higher than non-CCI children of the same age [Mdn = 3.2 (1.5–9)] vs. 1.6 [0.6–3.4] respectively, *p *= <0.001). PICU mortality in the CCI group was 7% (*n* = 62), compared to 2% in the non-CCI group (*n* = 225) (*p* < 0.001). Use of therapeutic limitations in the CCI group (3%) was higher than the non-CCI group (0.2%) (*p* < 0.001). Withholding treatment was more frequently used in non-CCI compared to CCI patients (76% vs. 43%, *p* < 0.001), while withdrawal of life-sustaining interventions was more widely adopted for the CCI patients compared to non-CCI patients (57% vs. 24%, *p* < 0.001).

### 3.2. Nursing workload and PICU resources use

The PICU resources use in both groups of CCI and non-CCI children are presented in Supplementary material Table S2. Clinical and statistical difference between groups is seen at admission, with higher nursing workload in CCI compared to non-CCI (Mdn = 27 [IQR = 18–34]; Mdn = 21 [IQR = 15–27] respectively, *p* < 0.001). CCI children experience more episodes of agitation (SAS score ≥5) per day than non-CCI (66% and 31% respectively; *p* < 0.001). Frequency and duration of utilization of PICU technologies were all statistically higher in the CCI group compared to non-CCI (*p* < 0.001). The proportion of isolation as an indicator of resources use was higher in CCI children compared to non-CCI (25% and 11% respectively, *p* < 0.001).

### 3.3. Risk factors of CCI in PICU

Risk factors of CCI in PICU children derived from the bivariate analysis resulted in eight variables included in the multivariate analysis: principal diagnosis, surgical interventions, standardized mortality risk score, ventilation support, type of PICU admissions, therapeutic limitations, agitation status and total NEMS. Results are shown in Supplementary material Table S3. Children with cardiac (aOR = 2.24; 95% CI = 1.68–2.98) and neurological diagnosis (aOR = 2.06; 95% CI = 1.58–2.69) were more likely to be CCI than patients with respiratory diagnosis. The adjusted odds ratios of children requiring cardiac, neurological, ENT, abdominal, and other surgeries were between 1.6 and 2.3 times higher than patients who did not require surgery. Patients using other types of ventilation than invasive and non-invasive were twice more likely to be CCI (aOR = 2.28; 95% CI = 1.84–2.82) than patients using invasive and non-invasive ventilation. Children with planned admissions (aOR = 0.40; 95% CI = 0.32–0.50) were less likely to be CCI than children with unplanned admissions. Children requiring therapeutic limitations were four times more likely to be CCI children (aOR = 4.50; 95% CI = 2.24–9.05). Children who were agitated (aOR = 1.87; 95% CI = 1.57–2.22) and very agitated (aOR = 3.71; 95% CI = 3.01–4.60) were more likely to be CCI than children with slight or no agitation. At PICU admission, predictive factors of CCI children were identified as following: cardiac diagnosis (aOR = 1.41; 95% CI = 1.09–1.84), surgical admission but particularly cardiac surgery (aOR = 5.58; 95% CI = 4.20–7.40), high mortality risk score (aOR = 1.26; 95% CI = 1.21–1.32), and unplanned admission (aOR = 0.32; 95% CI = 0.26–0.40).

## 4. Discussion

This retrospective national registry study is the first to report clinical characteristics and PICU resource use of CCI children, using a definition that includes both temporal and complexity criteria in a mixed PICU population in Switzerland. Key findings related to the prevalence rate for CCI children in PICU, the clinical characteristics and PICU resources needed of CCI children in the Swiss PICUs, and risk factors of CCI are discussed below.

Our CCI prevalence rate of 8% is relatively low, but sits within the reported prevalence range (6% to 14%) of long-stay patients (≥14 days) (4, 5). Our definition including complexity criteria (technology and healthcare resource use) allowed us to identify CCI children earlier than the commonly used threshold of 14 days. CCI patients in our study were admitted in mixed PICU/NICUs for a wide range of medical diagnosis seen in specialized PICU, thus our results should be transferrable to CCI term neonates and children admitted to these specialized PICUs, including neonatal, cardiac, general pediatric ICUs, and others.

The majority of the CCI clinical characteristics are similar to those reported in other studies with prolonged PICU LOS samples. CCI children in our study are young (median age <3 months) and have more cardiac conditions and surgeries than non-CCI children. Similar results were reported in other studies, with younger age (<12 months) being significant (4) and specific cardiac conditions being associated with long-stay PICU hospitalizations (20). We also found that CCI children experience more episodes of severe agitation than non CCI, and children with severe agitation episodes are almost four times more likely to be CCI. Although we were unable to document the sedatives administered in our study, we can assume that more episodes of severe agitation are likely to lead to more and prolonged sedation with a risk of developing iatrogenic delirium, which in turn can display signs of severe agitation This is coherent with findings of Patel et al, who described that long PICU technology use was associated with delirium development (8). Most CCI children are very young and particularly vulnerable, because their critical health conditions are likely to negatively impact their neurological development (21). Implementation of interventions to prevent delirium and withdrawal (22, 23) and to screen for potential neurodevelopmental sequelae (24) needs to be evaluated in this population.

CCI children in our sample had significantly higher risk of mortality compared to non-CCI. This result is not surprising considering the need for life-sustaining treatment for a prolonged period. Other studies reported comparable findings with mortality rates ranging between 8% and 20% in CCI patients with a PICU LOS greater than 14 days (4, 5). Our study showed that the use of therapeutic limitations was significantly higher in CCI children compared to non-CCI and this was confirmed by the significant association between therapeutic limitations use and CCI. Consistent with our results, a recent Swiss study reported that 62% of children with complex medical conditions died in the PICU setting and 96% of them died following withholding or withdrawal of life-sustaining interventions (17). These different results underline the importance of integrated supportive and intensive care models when caring for CCI patients to improve the quality of care (25, 26).

Our results also show that CCI children use a higher proportion of PICU resources than non CCI, which is consistent with international findings demonstrating a higher use of resources in CCI patients independent of the PICU LOS threshold used (5, 10, 27). When compared to children with a shorter PICU LOS, technology use of patients with prolonged PICU LOS was significantly higher for mechanical ventilation (18% vs. 81%, *p* < 0.001), vasoactive support (10% vs. 66%, *p* < 0.001), extracorporeal life support (0.1% vs. 6%, *p* < 0.001) and renal replacement therapy (1% vs. 22%, *p* < 0.001), respectively (27). We also found statistically significant differences in NEMS score in CCI patients compared with non-CCI patients, with little if no clinical relevance. This is probably due to our large sample size that can easy lead to statistical differences, but no clinical significance. Nevertheless, another study performed in a Swiss mixed PICU/NICU showed that medical residents’ perceived workload was strongly associated with patients’ LOS and nursing workload measured with NEMS (28). As CCI children have prolonged PICU LOS, our results at admission support the findings of the latter (34).

Risk factors for becoming a CCI child identified in our sample correspond with previous studies (11, 29). Although mechanical ventilation has already been shown to be an associated factor with the risk of becoming CCI (29, 30), our results highlight that patients with types of ventilation other than invasive and non-invasive are also at risk of CCI. This may be explained by the large amount of CCI children with chronic conditions who are hospitalized in PICU with pre-existing equipment (e.g., tracheostomy) (14, 31). Many other life-threatening conditions in previously healthy children, such as ARDS, nosocomial sepsis, to name a few, lead to prolonged PICU hospitalization and need at least one technology support. Thus, previously healthy children who may fully recover or not, are considered CCI children as per the definition in our study.

There are several limitations to this study. First, our results based on a Swiss data may not be generalized to CCI children from other countries, in which healthcare system delivery differ greatly based on Swiss data. Nevertheless, most of our findings were comparable to the literature from other Western countries adding to the validity of our results. Second, we were unable to identify children with repeated hospitalizations, also considered as CCI. It is thus possible that in our study, the reported number of CCI children is lower than the current reported prevalence rate of CCI children in Switzerland. Third, variables such as discharge with a device or a continuous infusion of a drug treatment as well as comorbidities, which would have been useful for the association with CCI development were not currently recorded in the national registry (11). Fourth, long-term survival or quality of life as outcome measures after PICU hospitalization would have been relevant to have a complete picture of the clinical characteristics of CCI patients (10). Fifth, such a large database easily leads to statistically significant differences, which must be put back into the clinical context to be interpreted with meaning.

## 5. Conclusion

Our study provides additional evidence highlighting the importance to consider CCI children being a central part of PICUs, given their specific characteristics and high resource needs when compared to non-CCI. It is, therefore, imperative for healthcare systems to adapt their model of care to best meet the specific needs of CCI children. Multidisciplinary PICU teams need to pay particular attention on the development and test early interventions to promote physical, cognitive, emotional, and social health of CCI children. The prevention of adverse events to enhance their QOL after PICU, and implementation research on patient- and family-centered care approach are necessary. Additional work is needed to understand which interventions during or following hospitalization reduce healthcare use with special attention to CCI children.

## Data Availability

The raw data supporting the conclusions of this article will be made available by the authors, without undue reservation.

## The OCToPuS consortium

Pediatric intensive care unit, Department Woman-Mother-Child, Lausanne University Hospital, Lausanne, Switzerland (**Anne-Laure Lauria**); Pediatric and neonatal intensive care unit, Department of Pediatrics, Gynecology and Obstetrics, University Hospitals of Geneva, Geneva, Switzerland (**Angelo Polito**; **Nathalie Bochaton**); Pediatric intensive care and pulmonology, University Children’s Hospital Basel UKBB, Basel, Switzerland (**Daniel Trachsel**; **Mark Marston**); Pediatric intensive care unit, Department of Children and Adolescents, University Hospital, Bern, Switzerland (**Silvia Schnidrig**, **Tilman Humpl**); Pediatric and neonatal intensive care unit, Children’s Hospital of Eastern Switzerland, St. Gallen, Switzerland (**Bjarte Rogdo**; **Ellen Wild**); Pediatric and neonatal intensive care unit, Department Children’s Hospital, Lucerne Cantonal Hospital, Switzerland (**Thomas Neuhaus**; **Sandra Stalder**); Department of neonatal and pediatric intensive care, University Children’s Hospital, Zurich, Switzerland (**Barbara Brotschi**; **Franziska von Arx**; **Anna-Barbara Schlüer**); Pediatric and neonatal intensive care unit, Department of Pediatrics, Cantonal Hospital Graubuenden, Chur, Switzerland (**Thomas Riedel**, **Pascale Van Kleef**).
