# Supplementary material for: Comparison of clinical characteristics and healthcare resource use of pediatric chronic and non-chronic critically ill patients in intensive care units: a retrospective national registry study
Source: Front Pediatr. 2023 Jun 26;11:1194833. doi: 10.3389/fped.2023.1194833 (PMC10331166; doi:10.3389/fped.2023.1194833)
Supplement: Supplementary file 1 [file Table1.docx]

**STROBE Statement**—Checklist of items that should be included in reports of ***cross-sectional studies***

|  | Item No | Recommendation | Page No |
| --- | --- | --- | --- |
| **Title and abstract** | 1 | (*a*) Indicate the study’s design with a commonly used term in the title or the abstract | 1 |
|  |  | (*b*) Provide in the abstract an informative and balanced summary of what was done and what was found | 2 |
| Introduction | | | |
| Background/rationale | 2 | Explain the scientific background and rationale for the investigation being reported | 3 |
| Objectives | 3 | State specific objectives, including any prespecified hypotheses | 3 |
| Methods | | | |
| Study design | 4 | Present key elements of study design early in the paper | 4 |
| Setting | 5 | Describe the setting, locations, and relevant dates, including periods of recruitment, exposure, follow-up, and data collection | 4 |
| Participants | 6 | (*a*) Give the eligibility criteria, and the sources and methods of selection of participants | 4 |
| Variables | 7 | Clearly define all outcomes, exposures, predictors, potential confounders, and effect modifiers. Give diagnostic criteria, if applicable | 4-5  Table 1 |
| Data sources/ measurement | 8* | For each variable of interest, give sources of data and details of methods of assessment (measurement). Describe comparability of assessment methods if there is more than one group | 4-5 |
| Bias | 9 | Describe any efforts to address potential sources of bias | N/A |
| Study size | 10 | Explain how the study size was arrived at | 4 |
| Quantitative variables | 11 | Explain how quantitative variables were handled in the analyses. If applicable, describe which groupings were chosen and why | 4-5 |
| Statistical methods | 12 | (*a*) Describe all statistical methods, including those used to control for confounding | 5-6 |
|  |  | (*b*) Describe any methods used to examine subgroups and interactions | 5-6 |
|  |  | (*c*) Explain how missing data were addressed | N/A |
|  |  | (*d*) If applicable, describe analytical methods taking account of sampling strategy | N/A |
|  |  | (*e*) Describe any sensitivity analyses | N/A |
| Results | | | |
| Participants | 13* | (a) Report numbers of individuals at each stage of study—eg numbers potentially eligible, examined for eligibility, confirmed eligible, included in the study, completing follow-up, and analysed | N/A |
|  |  | (b) Give reasons for non-participation at each stage | N/A |
|  |  | (c) Consider use of a flow diagram | N/A |
| Descriptive data | 14* | (a) Give characteristics of study participants (eg demographic, clinical, social) and information on exposures and potential confounders | 6-7, Table S1 |
|  |  | (b) Indicate number of participants with missing data for each variable of interest | N/A |
| Outcome data | 15* | Report numbers of outcome events or summary measures | 6-7, Tables 1, 2, 3 and S1 and S2 |
| Main results | 16 | (*a*) Give unadjusted estimates and, if applicable, confounder-adjusted estimates and their precision (eg, 95% confidence interval). Make clear which confounders were adjusted for and why they were included | N/A |
|  |  | (*b*) Report category boundaries when continuous variables were categorized | Table 1 |
|  |  | (*c*) If relevant, consider translating estimates of relative risk into absolute risk for a meaningful time period | N/A |
| Other analyses | 17 | Report other analyses done—eg analyses of subgroups and interactions, and sensitivity analyses | N/A |
| Discussion | | | |
| Key results | 18 | Summarise key results with reference to study objectives | 7-8 |
| Limitations | 19 | Discuss limitations of the study, taking into account sources of potential bias or imprecision. Discuss both direction and magnitude of any potential bias | 9 |
| Interpretation | 20 | Give a cautious overall interpretation of results considering objectives, limitations, multiplicity of analyses, results from similar studies, and other relevant evidence | 9-10 |
| Generalisability | 21 | Discuss the generalisability (external validity) of the study results | 8 |
| Other information | | | |
| Funding | 22 | Give the source of funding and the role of the funders for the present study and, if applicable, for the original study on which the present article is based | 12 |

| **Table S1. Comparison of demographics and clinical characteristics between CCI and non-CCI groups** | | | | | |
| --- | --- | --- | --- | --- | --- |
| **Characteristics** | | **Total PICU Sample**  (*N =* 12 375) (100%) | **CCI^a^ patients**  (*n* = 982) (8%) | **Non-CCI^b^ patients**  (*n* = 11 393) (92%) | ***p*-value^c^** |
| PICU length of stay, *Mdn [IQR]* | | 1.8 [1-5] | 12 [9-19] | 1.6 [1-4] | <0.001 |
| Age [months]*, Mdn [IQR]* | | 6.2 [0-60] | 2.8 [0-36] | 6.7 [0-60] | <0.001 |
| Males, *n (%)* | | 7 108 (57) | 595 (61) | 6 513 (57) | 0.037 |
| Unplanned admissions, *n (%)* | | 8 696 (70) | 750 (76) | 7 946 (70)] | <0.001 |
| PICU readmissions (<48h00), *n (%)* | | 144 (1) | 13 (1) | 131 (1) | 0.626 |
| Provenance, *n (%)* | |  |  |  | <0.001 |
|  | Operating room | 6 040 (49) | 432 (44) | 5 608 (49) |  |
|  | Emergency department | 2 116 (17) | 161 (16) | 1 955 (17) |  |
|  | Ward | 1 770 (14) | 149 (15) | 1 621 (14) |  |
|  | Intermediate care, recovery room | 239 (2) | 29 (3) | 210 (2) |  |
|  | Other | 2 206 (18) | 208 (21) | 1 998 (18) |  |
| Destination, *n (%)* | |  |  |  | <0.001 |
|  | Ward | 8 119 (66) | 553 (56) | 7 566 (66) |  |
|  | Intermediate care | 2 777 (23) | 226 (23) | 2 551 (22) |  |
|  | Home | 575 (5) | 73 (7) | 502 (4) |  |
|  | Other PICU | 243 (2) | 21 (2) | 222 (2) |  |
|  | Other | 661 (5) | 109 (11) | 552 (5) |  |
| Principal diagnosis, *n (%)* | |  |  |  | <0.001 |
|  | Respiratory | 3 825 (31) | 275 (28) | 3 550 (31) |  |
|  | Cardiac | 1 556 (13) | 231 (24) | 1 325 (12) |  |
|  | Neurological | 1 527 (12) | 133 (14) | 1 394 (12) |  |
|  | Injury | 1 431 (12) | 80 (8) | 1 351 (12) |  |
|  | Prematurity | 722 (6) | 16 (2) | 706 (6) |  |
|  | Sepsis | 262 (2) | 43 (4) | 219 (2) |  |
|  | Oncology | 172 (1) | 10 (1) | 163 (1) |  |
|  | Other | 2 879 (23) | 194 (20) | 2 685 (24) |  |
| Surgical interventions, *n (%)* | | *N = 3 938 (32%)* |  |  | <0.001 |
|  | Cardiac | 1 099 (28) | 198 (47) | 901 (26) |  |
|  | Abdominal | 686 (17) | 64 (15) | 622 (18) |  |
|  | Ear, Nose, Throat (ENT) | 688 (17) | 49 (12) | 639 (18) |  |
|  | Neurological | 497 (13) | 46 (11) | 451 (13) |  |
|  | Post-procedure monitoring | 354 (9) | 28 (7) | 326 (9) |  |
|  | Orthopaedic | 234 (6) | 6 (1) | 228 (7) |  |
|  | Thoracic | 210 (5) | 30 (7) | 180 (5) |  |
|  | Craniofacial | 170 (5) | 0 (0) | 170 (5) |  |
| Mortality risk score^d^ | |  |  |  |  |
|  | CRIB II, *Mdn [IQR] (n = 1 361)* | 2 [1-5] | 3 [1.4-12] | 2 [1-5] | 0.005 |
|  | PIM2, *Mdn [IQR] (n = 10 702)* | 1.7 [0.8-3.8] | 3.2 [1.5-9] | 1.6 [0.6-3.4] | <0.001 |
|  | SAPS, *Mdn [IQR] (n = 265)* | 10 [3-19] | 15 [7-28] | 10 [3-18] | 0.029 |
| Mortality, *n (%)* | | 287 (2) | 62 (7) | 225 (2) | <0.001 |
| Therapeutic limitations use, *n (%)* | | 55 (0.4) | 30 (3) | 25 (0.2) | <0.001 |
|  | Withholding treatment, *n (%)* | 32 (58) | 13 (43) | 19 (76) | <0.001 |
|  | Withdrawal of life-sustaining interventions, *n (%)* | 23 (42) | 17 (57) | 6 (24) | <0.001 |

^a^CCI, chronically critically ill; ^b^Non-CCI, non-chronically critically ill

^c^CCI vs non-CCI group comparison using Wilcoxon-Mann-Whitney or χ2 test as appropriate

^d^CRIB II: Clinical Risk Index for Babies. PIM2: Paediatric Index of Mortality. SAPS: Simplified Acute Physiology Score

| **Table S2. Comparison of Nursing workload and PICU resources use between CCI and non-CCI groups** | | | | | | |
| --- | --- | --- | --- | --- | --- | --- |
| **Characteristics** | | | **Total PICU Sample**  (*N =* 12 375) (100%) | **CCI patients^a^**  (*n* = 982) (8%) | **Non-CCI patients^b^**  (*n* = 11 393) (92%) | ***p*-value**^c^ |
| NEMS score^d^ | | |  |  |  |  |
|  | At admission, *Mdn [(IQR]* | | 21 [15-27] | 27 [18-34] | 21 [15-27] | <0.001 |
|  | At discharge, *Mdn [IQR]* | | 15 [9-18] | 15 [9-18] | 15 [9-18] | <0.001 |
|  | Total score, *Mdn [IQR]* | | 21 [17-26] | 22 [17-27] | 21 [16-26] | <0.001 |
| Agitation status per day^e^ | | |  |  |  |  |
|  | Agitated status, *n (%)* | | 4 179 (34) | 647 (66) | 3 532 (31) | <0.001 |
|  | Very agitated status, *n (%)* | | 1 001 (8) | 282 (29) | 719 (6) | <0.001 |
| Technologies use | | |  |  |  |  |
|  | Intravenous medications*, n (%)* | | 10 868 (88) | 950 (97) | 9 918 (87) | <0.001 |
|  | | Median duration, *days [IQR]* | 2 [1-4] | 10 [7-15] | 1 [1-3] | <0.001 |
|  | Invasive and non-invasive ventilation*, n (%)* | | 5 969 (48) | 830 (85) | 5 139 (45) | <0.001 |
|  | | Median duration, *days [IQR]* | 1 [1-4] | 7 [4-11] | 1 [1-3] | <0.001 |
|  | Other ventilation*, n (%)* | | 6 033 (49) | 771 (79) | 5 262 (46) | <0.001 |
|  | | Median duration, *days [IQR]* | 1 [1-2] | 3 [1-6] | 1 [0-2] | <0.001 |
|  | Multiple vasoactive medications*, n (%)* | | 1 405 (11) | 411 (42) | 994 (9) | <0.001 |
|  | | Median duration, *days [IQR]* | 1 [1-3] | 3 [2-6] | 1 [1-2] | <0.001 |
|  | Dialysis, *n (%)* | | 147 (1) | 78 (8) | 69 (1) | <0.001 |
|  | | Median duration, *days [IQR]* | 4 [1-7] | 6 [3-11] | 2 [1-4] | <0.001 |
|  | ECMO^f^*, n (%)* | | 63 (1) | 42 (4) | 21 (0) | <0.001 |
|  | | Median duration, *days [IQR]* | 5 [2-9] | 7 [4-12] | 2 [0-4] | <0.001 |
| Healthcare resources use | | |  |  |  |  |
|  | Isolation, *n (%)* | | 1 469 (12) | 245 (25) | 1 224 (11) | <0.001 |
|  | | Median duration, *days [IQR]* | 2 [1-4] | 8 [3-12] | 2 [1-3] | <0.001 |

^a^CCI, chronically critically ill; bNon-CCI, non-chronically critically ill

^c^CCI vs non-CCI group compared by Wilcoxon-Mann-Whitney or χ2 test as appropriate

^d^Nine Equivalents of Nursing Manpower use Score

^e^Agitation was measured each day using the Sedation Agitation Score (SAS) and determined by a AS score of 5 (agitated) or 6 (very agitated)

^f^Extracorporeal Membrane Oxygenation therapy

| **Table S3. Risk factors of chronic critical illness in PICU children, by a bivariable and a multivariable logistic regression** | | | | | | | | | |  |
| --- | --- | --- | --- | --- | --- | --- | --- | --- | --- | --- |
|  | | ***Bivariable analysis*** | | | ***Multivariable models*** | | | | | |
|  | | | | | **Associated factors** | | | **Predictors** | | |
|  | | **OR (SE)** | **95% CI** | **p** | **aOR (SE)** | **95% CI** | **p** | **aOR (SE)** | **95% CI** | **p** |
| Age | |  |  |  |  |  |  |  |  |  |
|  | ≤1 year old | - | reference | - | - | - | - | - | - | - |
|  | >1 year old | 0.727 (0.20) | 0.425-1.243 | 0.245 | - | - | - | - | - | - |
| Principal diagnosis | |  |  |  |  |  |  |  |  |  |
|  | Respiratory | - | reference | - | - | reference | - | - | reference | - |
|  | Cardiac | 2.250 (0.70) | 1.225-4.135 | 0.009 | 2.241 (0.33) | 1.682-2.985 | <0.001 | 1.414 (0.19) | 1.090-1.835 | 0.009 |
|  | Neurological | 1.231 (0.24) | 0.847-1.791 | 0.275 | 2.062 (0.28) | 1.580-2.691 | <0.001 | 1.110 (0.14) | 0.874-1.410 | 0.395 |
|  | Other | 0.864 (0.08) | 0.716-1.043 | 0.127 | 1.110 (0.12) | 0.900-1.370 | 0.328 | 0.811 (0.07) | 0.678-0.970 | 0.021 |
| Surgical interventions | |  |  |  |  |  |  |  |  |  |
|  | No surgery | - | reference | - | - | reference | - | - | reference | - |
|  | Cardiac | 3.085 (1.16) | 1.478-6.440 | 0.003 | 2.391 (0.38) | 1.748-3.270 | <0.001 | 5.580 (0.80) | 4.205-7.401 | <0.001 |
|  | Neurological | 1.431 (0.38) | 0.856-2.397 | 0.172 | 1.918 (0.37) | 1.315-2.800 | <0.001 | 2.102 (0.37) | 1.484-2.980 | <0.001 |
|  | Ear, Nose, Throat | 1.077 (0.61) | 0.356-3.255 | 0.896 | 2.228 (0.42) | 1.546-3.211 | <0.001 | 1.956 (0.32) | 1.416-2.700 | <0.001 |
|  | Abdominal | 1.445 (0.54) | 0.697-2.993 | 0.332 | 1.993 (0.34) | 1.433-2.773 | <0.001 | 2.455 (0.36) | 1.834-3.286 | <0.001 |
|  | Other | 0.994 (0.25) | 0.610-1.619 | 0.980 | 1.662 (0.28) | 1.200-2.304 | <0.001 | 1.921 (0.27) | 1.446-2.553 | <0.001 |
| Standardized mortality risk score | | 1.299 (0.02) | 1.258-1.341 | <0.001 | 1.074 (0.04) | 1.008-1.145 | 0.003 | 1.264 (0.03) | 1.209-1.321 | <0.001 |
| Ventilation support | |  |  |  |  |  |  |  |  |  |
|  | Invasive and non-invasive ventilation | - | reference | - | - | reference | - | - | - | - |
|  | Other ventilation | 2.641 (0.60) | 1.689-4.131 | <0.001 | 2.278 (0.25) | 1.840-2.822 | <0.001 | - | - | - |
|  | No ventilation support | 0.342 (0.09) | 0.203-0.575 | <0.001 | 0.747 (0.12) | 0.540-1.033 | 0.080 | - | - | - |
| Type of PICU admission | |  |  |  |  |  |  |  |  |  |
|  | Unplanned | - | reference | - | - | reference | - | - | reference | - |
|  | Planned | 0.713 (0.17) | 0.445-1.142 | 0.160 | 0.400 (0.05) | 0.320-0.501 | <0.001 | 0.319 (0.03) | 0.260-0.392 | <0.001 |
